# A high performance cost-effective digital complex correlator for an X-band polarimetry survey

**DOI:** 10.1186/s40064-016-2109-5

**Published:** 2016-04-19

**Authors:** Miguel Bergano, Armando Rocha, Luís Cupido, Domingos Barbosa, Thyrso Villela, José Vilas Boas, Graça Rocha, George F. Smoot

**Affiliations:** Department of Electronics, Telecommunication and Informatics (DETI), Instituto de Telecomunicações, University of Aveiro, Campus Universitário de Santiago, 3810-193 Aveiro, Portugal; Department of Electronics, Telecommunication and Informatics (DETI), University of Aveiro, Campus Universitário de Santiago, 3810-106 Aveiro, Portugal; LC-Technologies, Aveiro, Portugal; Instituto de Telecomunicações, Campus Universitário de Santiago, 3810-193 Aveiro, Portugal; Instituto Nacionals de Pesquisas Espaciais (INPE) - Divisão de Astrofísica – DAS, Av. dos Astronautas, 1.758, Jd. Granja, CEP 12227-010 São Jose dos Campos, SP Brazil; Jet Propulsion Laboratory, M/S 169-327, 4800 Oak Grove Drive, Pasadena, CA 91109 USA; Caltech, Cahill Building MS 59-33 1200 E. California, Pasadena, CA 91125-3300 USA; Lawrence Berkeley National Lab, 1 Cyclotron Road, MS 50-5005, Berkeley, CA 94720 USA; Université Paris-Diderot APC, Bâtiment Condorcet, 10 rue Alice Domon et Léonie Duquet, 75205 Paris Cedex 13, France

**Keywords:** Radioastronomy (astronomy, astrophysics and cosmology), Logic design (hardware), Register-transfer-level implementation (hardware)

## Abstract

The detailed knowledge of the Milky Way radio emission is important to characterize galactic foregrounds masking extragalactic and cosmological signals. The update of the global sky models describing radio emissions over a very large spectral band requires high sensitivity experiments capable of observing large sky areas with long integration times. Here, we present the design of a new 10 GHz (X-band) polarimeter digital back-end to map the polarization components of the galactic synchrotron radiation field of the Northern Hemisphere sky. The design follows the digital processing trends in radio astronomy and implements a large bandwidth (1 GHz) digital complex cross-correlator to extract the Stokes parameters of the incoming synchrotron radiation field. The hardware constraints cover the implemented VLSI hardware description language code and the preliminary results. The implementation is based on the simultaneous digitized acquisition of the Cartesian components of the two linear receiver polarization channels. The design strategy involves a double data rate acquisition of the ADC interleaved parallel bus, and field programmable gate array device programming at the register transfer mode. The digital core of the back-end is capable of processing 32 Gbps and is built around an Altera field programmable gate array clocked at 250 MHz, 1 GSps analog to digital converters and a clock generator. The control of the field programmable gate array internal signal delays and a convenient use of its phase locked loops provide the timing requirements to achieve the target bandwidths and sensitivity. This solution is convenient for radio astronomy experiments requiring large bandwidth, high functionality, high volume availability and low cost. Of particular interest, this correlator was developed for the Galactic Emission Mapping project and is suitable for large sky area polarization continuum surveys. The solutions may also be adapted to be used at signal processing subsystem levels for large projects like the square kilometer array testbeds.

## Background

The plethora of radio and microwave experiments exploring large radio spectral bands from the Megahertz to many dozens of Gigahertz promise a very high science impact on galactic and extragalactic radio astronomy (RA). The concurring radio surveys planned with many instruments, including those to be performed as parts of Key Science projects of the square kilometer array (SKA) and its pathfinders and precursors require the refining of the radio global sky models (GSM) and knowledge improvement of galactic foreground radiation. This improvement enables a better separation of the different emission critical components to the analysis of the cosmic microwave background (CMB) observations obtained by ground and space missions above 70 GHz (Komatsu et al. 2011; Planck Collaboration 2013). Furthermore, it allows a better separation of low frequency components below 1.4 GHz from observations obtained with radio-interferometers like the SKA and its pathfinders precursors (LOFAR, MWA, MeerKAT, ASKAP). The necessary high sensitivity large scale mapping in radio requires long observations over large sky areas with exquisite control of receptors systematics. The recent advances in digital radio technologies have raised receiver design to a new level enabling much better systematics control and information processing. Sampling at radio-frequency (RF) and replacing RF and intermediate frequency (IF) analog hardware by digital component subsystems, improves the performance and robustness making it immune to RFI (Johnson et al. 2006). Nowadays, digital converters with rates above 1 GSps and field programmable logic devices, capable of parallel digital signal processing at high rates, are widely available and affordable. However, reduction of size, cost and hardware complexity are an advantage at the expense of a more demanding digital design.

The use of digital solutions for applications such as radiometry and polarimetry, carries additional advantages over analog designs. Stability and lower gain fluctuations are advantages that offer more reliability and easier calibration of instruments (Gaier et al. 2012; Gasiewski and Kunkee 1993).

Historically, in 1961 Weinreb (Weinreb 1961) proposed the first single bit radiometer based on a 1 bit digital converters (ADC), a tapped single bit shift register as a delay line and an exclusive or as a multiplier. The solution offered enough sensitivity (64 % of a perfect analog correlator) and paved the way for much of the developments of digital correlator implementations (Bischoff et al. 2013; Cleary 2010; Padin et al. 1993; Piepmeier and Gasiewski 2001). More recently, the collaboration for astronomy signal processing and electronic research (CASPER) and the UNIBOARD project (Parsons et al. 2006; Szomoru 2011; Werthimer 2011) represent an evolution of digital technology applied to radio astronomy. The UNIBOARD uses a complex and expensive system with several Altera Stratix IV FPGAs connected by high-speed links, with a 4 GHz input bandwidth providing 64 output bands. Less demanding applications may however suffer from the CASPER or UNIBOARD solution complexity and its higher cost. A recent paper (Holler et al. 2012) compares the cost of a specific implementation of an analog correlator with a digital equivalent one using CASPER developed boards.

Correlation receivers and polarimeters are extensively described in (Rohlfs and Wilson 2004). We apply it to the development of a new 10 GHz polarimeter, with 1 GHz bandwidth, hence we achieve five times more bandwidth for large sky area polarimetry surveys. This solution uses standards ADCs and an Altera Cyclone III FPGA providing a very cost-effective performance.

## Design and method

### Polarimeter back-end application scenario

The radio mapping of large sky areas, in the frequency range of 50 MHz to 1 GHz, is important to update the GSM and offer a database of legacy templates for future astrophysical use. The mapping of the polarized emission between 5 and 10 GHz projects is the target of several projects such as the Galactic Emission Mapping project (GEM) (Torres et al. 1996) and the more recent C-Band All Sky Survey (CBASS) project (King et al. 2010) to improve the polarized foreground subtraction obtained from CMB missions. The GEM project began to map the polarized microwave emission of the sky in Brazil, having a counterpart in the Northern Hemisphere, after the installation of an antenna in Portugal (Fonseca et al. 2006). This combination of observations will cover about 85 % of the sky.

**Fig. 1:**
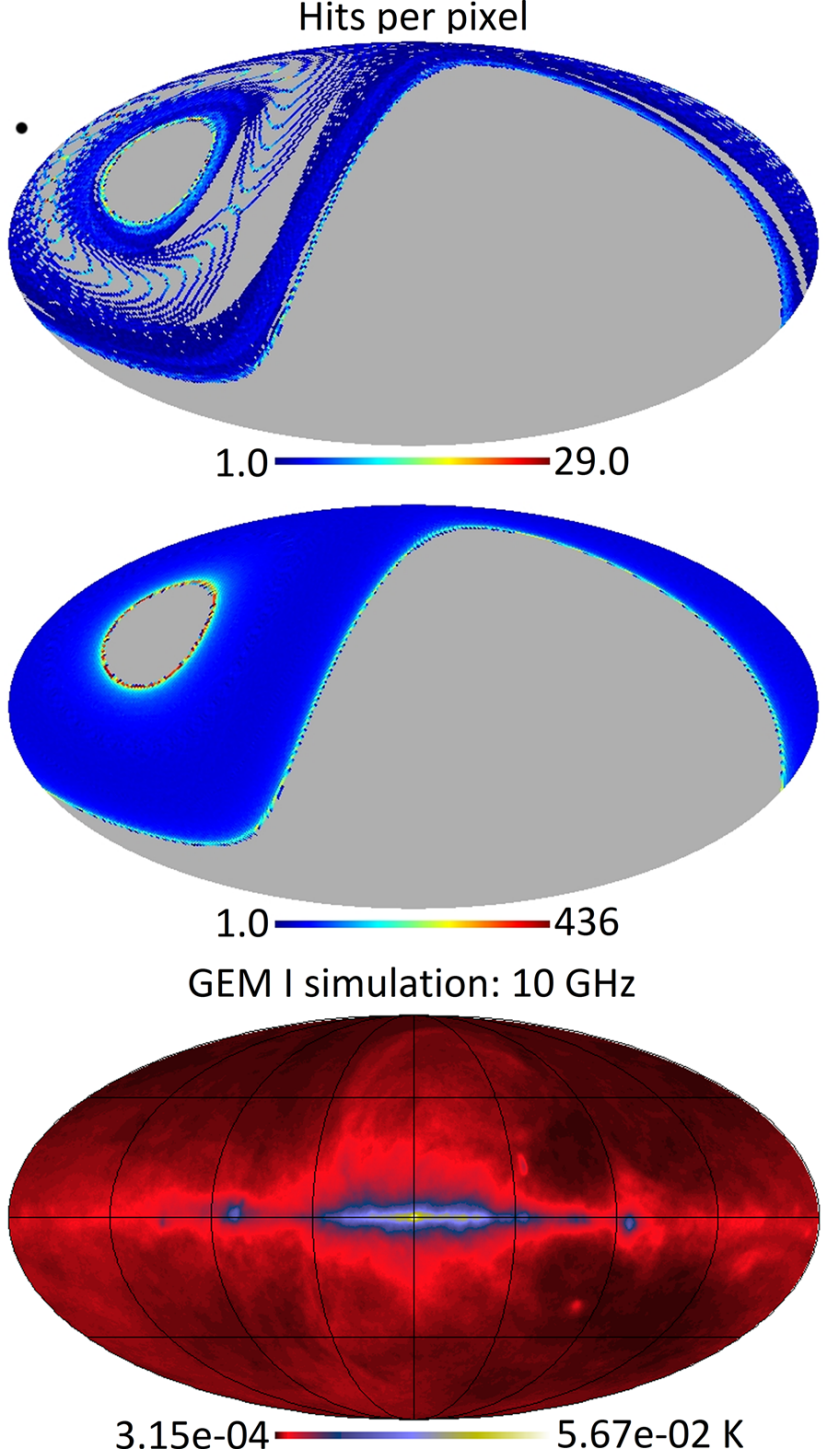
Simulation of scanning strategy for the Northern GEM survey. From *top* to *bottom* simulated 10 GHz total flux, hits/pixel for 1 h continuous observation; hits/pixel for 1 day continuous observations and hits/pixel for 1 day continuous observations

Figure 1 represents an illustration of the Northern hemisphere scanning strategy and the 10 GHz all-sky template obtained after extrapolation of GSM templates from (Giardino et al. 2002). The Fiducial values on sky flux for receiver and mapping strategy design were obtained from the simulated sky at 10 GHz and incorporating the radio synchrotron spectral index corrections after (Planck Collaboration et al. 2015; Giardino et al. 2002). Initiated as part of the GEM collaboration and building on the lessons of the C-band (5 GHz) polarimeter (Bergano et al. 2007) here we outline the X-band (10 GHz) new digital design. The foreground removal at lower frequencies requires the measurement of sky noise temperature with a resolution of a few micro Kelvin. To enhance the sensitivity and reduce the sky survey integration time to acceptable values, the bandwidth must be at least 1 GHz or higher.

### Polarimeter analog hardware

**Fig. 2:**
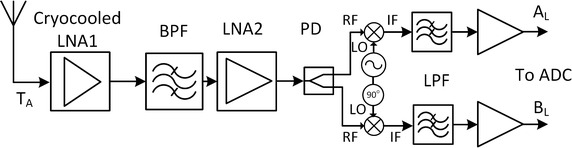
Front-end block diagram for the left channel depicting the Cartesian components A_L_ and B_L_ for the left circular polarization

The polarimeter sensitivity and dynamic range depend on antenna, system noise and integration time, thus influencing the digital design of the backend, where final detection of Stokes parameters takes place. The expected polarized signal power equivalent temperature at 10 GHz is ~0.1 mK so a long integration time is needed. The antenna is a 9 m Cassegrain type with an HPBW ϴ_3dB_ = 0.28° and equipped with a corrugated feed horn. A polarizer followed by an orthomode transducer (OMT) carries the separation between left and right circular polarizations (LHCP and RHCP). Both polarization channels are further amplified until they are digitized. The analog front end block diagram of the receiver for one channel is depicted in Fig. 2. In short, the analog chain is responsible for amplifying the signals to the required digital entry levels. Since the galactic synchrotron emission is linearly polarized, we designed the polarimeter to output the linear U and V Stokes parameters through the correlation of the incoming circular polarization components and thus minimize calibration systematics and output Stokes parameters.

#### The analog receiver chain

The signal in Fig. 1 is amplified using a low noise amplifier (LNA) (LNC7-10A from Low Noise Factory) operating at 70 K inside a Dewar. A low loss 1 GHz bandwidth bandpass waveguide filter labelled (BPF) performs the first stage filtering to remove interferences and reduce the input power at the following amplifying stages. The following amplifying stage labelled (LNA2) uses ultra-low noise pHEMTs and mHEMTs technology devices. Finally, a conversion to baseband, complex in-phase and quadrature components, is performed and followed by 500 MHz bandwidth low-pass filters labelled (LPF) and amplifiers. Each Cartesian component is digitized by one ADC.

The gain/attenuation budget on Table 1 summarizes the relevant system parameters. The system noise temperature T_SYS_ includes contributions from the antenna—T_A_ (for T_A_ was assumed a value of 10 K), sky—T_SKY_ (for T_SKY_—atmosphere, spillover, was assumed a value of 5 K) and receiver—T_REC_ (for T_REC_ was assumed a value of 15 K) temperatures: T_SYS_ = T_A_ + T_SKY_ + T_REC_. The polarimeter design main guidelines take into account the following specifications:

**Table 1:** Gain/attenuation contributions along the receiver

	T_SYS_	LNA	Filter	2nd LNA	Power splitter	Mixer	LPF	Amp.	ADC
T_eq_ (K)	30.0	3.0	438.4	20.7	288.6	1539.8	288.6	359.2	
NF (dB)		0.04	4.0	0.3	3.0	8.0	3.0	3.5	
Gain (dB)		35.0	−4.0	15.0	−3.0	−8.0	−3.0	52.0	
Power (dBm)	−93.8	−58.8	−62.8	−47.8	−50.8	−58.8	−61.8	−9.8	−11.0

- RF center frequency: 10 GHz;
- RF bandwidth B: 1 GHz;
- Polarimeter noise temperature T_SYS_: 30 K;
- Resolution: ΔT = 0.1 mK (1st Stokes parameter).

The polarimeter is designed to deliver N estimations (pixels) of the Stokes parameters within the antenna HPBW with N = 10 a typical value. Using the antenna HPBW and the revolutions per minute ω_rot_ the sky pixel surveying rate, N_P_ (points/s), is given by:

1 $$N_{P} = \frac{6N}{{\theta_{3dB} }}\omega_{Rot}$$

Assuming N = 10 and ω_rot_ = 1 rpm then N_P_ is about 220. The pixel integration time τ_p_, within the digital backend, is given by:

2 $$\tau_{P} = \frac{1}{{N{}_{P}}}$$

and, for the above operating parameters, τ_p_ = 4.66 ms. The well-known radiometer equation can now be used to estimate the full integration time τ to achieve the target resolution for the first Stokes parameter (I) at each surveyed pixel:

3 $$\tau = \frac{1}{2B}\left( {\frac{{T_{SYS} }}{\Delta T}} \right)^{2}$$

Considering 1 GHz bandwidth B, T_SYS_ = 30 K and ΔT = 1 mK the total integration time is τ = 45 s. The total surveying time *τ*_Survey_ (in days) is given by:

4 $$\tau_{Survey} = \frac{\tau }{{1440\tau_{P} }}$$

where 1440 is the number of revolutions per day, so *τ*_Survey_ is about 7 days. The larger the bandwidth B and the smaller the system temperature T_SYS_ the shorter will be measurement campaign duration.

The calculation of the Stokes parameters follows the work developed in (Bergano et al. 2011).

5 $$I = \left\langle {\left( {A_{L} + iB_{L} } \right) \cdot \left( {A_{L} - iB_{L} } \right) + \left( {A_{R} + iB_{R} } \right) \cdot \left( {A_{R} - iB_{R} } \right)} \right\rangle = A_{L}^{2} + B_{L}^{2} - A_{R}^{ 2} - B_{R}^{ 2}$$

6 $$Q = \left\langle {\left( {A_{L} + iB_{L} } \right) \cdot \left( {A_{L} - iB_{L} } \right) - \left( {A_{R} + iB_{R} } \right) \cdot \left( {A_{R} - iB_{R} } \right)} \right\rangle = A_{L}^{2} + B_{L}^{2} - A_{R}^{ 2} - B_{R}^{ 2}$$

7 $$U = 2 {\text{Re}}\left\langle {\left( {A_{L} - iB_{L} } \right) \cdot \left( {A_{R} + iB_{R} } \right)} \right\rangle = 2\left( {A_{L} A_{R} + B_{L} B_{R} } \right)$$

8 $$V = 2 {\text{Im}}\left\langle {\left( {A_{L} - iB_{L} } \right) \cdot \left( {A_{R} + iB_{R} } \right)} \right\rangle = 2\left( {A_{L} B_{R} - A_{R} B_{L} } \right)$$

where A_R_, A_L_, B_R_ and B_L_ are the in-phase (A) and quadrature (B) components for the right and left channels complex amplitude and 〈〉 represents the time average. Analyzing (5) to (8) we can see that a direct implementation requires 8 multipliers and 8 adders. The Stokes parameters resolution depends on the system equivalent noise temperature (T_SYS_), detection bandwidth (B) and integration time (*τ*_Survey_). The first integration is made within the field programmable gate array (FPGA) (Bergano et al. 2011) and the final one by the host acquisition computer.

### Digital polarimeter baseline design

**Fig. 3:**
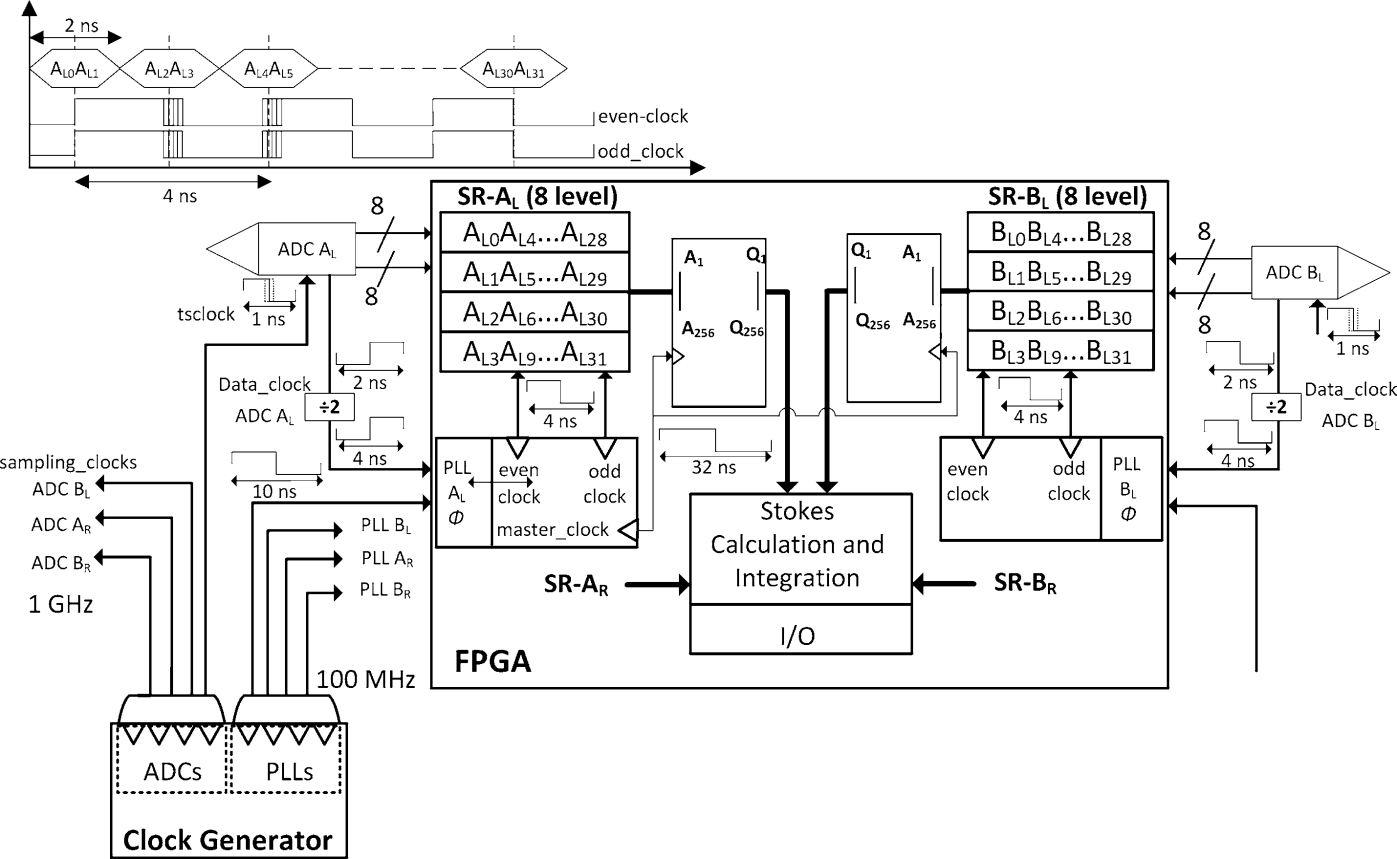
General block diagram of the polarimeter implementation

The block diagram of the digital receiver backend is shown in Fig. 3. This diagram begins with a standard FPGA clocked at 250 MHz, a clock generator and ADCs with two parallel data buses. It details only, for the sake of simplicity, the acquisition of left channel Cartesian components (A_L_ and B_L_) and the system clock derivation scheme. The required strategies are reached by applying dedicated techniques, namely:

- Two 8 bit ADCs for digitizing each channel Cartesian components (total of four ADCs);
- ADCs with interleaved data buses providing two samples per clock period (presenting 500 MSps per ADC bus to the FPGA);
- Double data rate interfacing to the FPGA, sampling at 500 MSps from the 250 MHz clock.

The ADC achieves 1 GSPS bandwidth by exploiting 1:2 demultiplexing in order to produce two 8 bit streams of samples at half the sample rate. The acquisition of the ADC samples is performed along with its data-clock (500 MHz) divided by two, i.e 250 MHz. Figure 3 shows the two ADC streams feeding four shift registers each 8 stages long. Both the rising and the falling edges of the clocks (250 MHz) are used to clock the samples into the proper shift register at the proper time.

A clock generator chip provides a 1 GHz sampling clock (t_Sclock_) for each of the four ADCs (ADC A_L_ to ADC B_R_). The FPGA phase lock loops (PLL) allow phase adjustable clocks at 250 MHz (even clock and odd clock) that are used to latch the Cartesian component samples to a set of shift registers (SR-A_L_ to SR-B_R_) and derives, as well, a 31.25 MHz clock (master_clock) to clock the Stokes parameters calculation blocks. The 8 bit samples A_L0_A_L4_…AL_28_, presented at one of the ADC buses, are latched to the first shift register by the ascending transition of the even_clock (4 ns). The simultaneously available samples (A_L1_, A_L5_…AL_29_) at the other ADC bus, are latched to another shift register by the ascending transition of the odd_clock. The samples A_L2_A_L6_…AL_30_ and A_L3_A_L7_…AL_31_ are stored in the next shift registers with the same strategy but now using the descending transition of the two clocks.

The same solution is implemented to handle the quadrature component of the left channel B_L_ (partially depicted in Fig. 3), as well as the Cartesian components for the right channel (A_R_ and B_R_). The four shift registers now hold 32 ns time series of all the receiver Cartesian components sampled at 1 GSps. After 32 samples have been clocked into the shift registers, all 32 8 bit samples are loaded into a 256-bit register. Further processing will be carried out in blocks clocked by a second clock domain.

The parallel calculation and accumulation of 32 samples of the Stokes parameters (1–4) I, Q, U and V is performed each 32 ns in the Stokes calculation block and integrated in a 22 bits register length. A set of 2N_S_ samples of the integrated Stokes parameters are further integrated in the accumulators (ACC) with 36 bits register length and results are truncated to 24 bits that are enough to resolve 0.1 mK over 30 K (of T_SYS_) for the 1st Stokes parameter. Finally, a Multiplexer (MUX)/First In First Out Memory (FIFO) holds the integrated data to be sent to the host computer and performs the data transfer protocol.

The data transfer rate from the FPGA to the host computer determines the design of the I/O hardware and in particular buffer sizes. The data rate R_P_ (bps) to transmit all the 4 Stokes parameters (each 3 bytes long) is given by:

9 $$R_{P} = 96N_{P}$$

with N_P_ provided by (1). The 21 Kbps transfer rate can be handled by any standard parallel or serial bus.

The delays at ADC bit level, from the FPGA I/O pin to the input shift registers must be well known and equalized and an adequate time alignment of the sampling clock (t_Sclock_) of the ADC with the FPGA internal latching clock must be achieved. The FPGA must be programmed at a register transfer level to assign the I/O pins and route all the bits to the shift registers with predictable delays. Also, the latching clocks must have an adjustable phase. The digital hardware design requirements are summarized below:

- Reduced time dispersion from I/O pin to input registers;
- ADC located strategically close to the FPGA dedicated PLLs;
- Adequate clock distribution and FPGA PLLs programming.

We now describe the hardware parts selection strategy and perform an analysis of the clock and timing requirements for the proposed solution.

## Experimental measurements and results

**Fig. 4:**
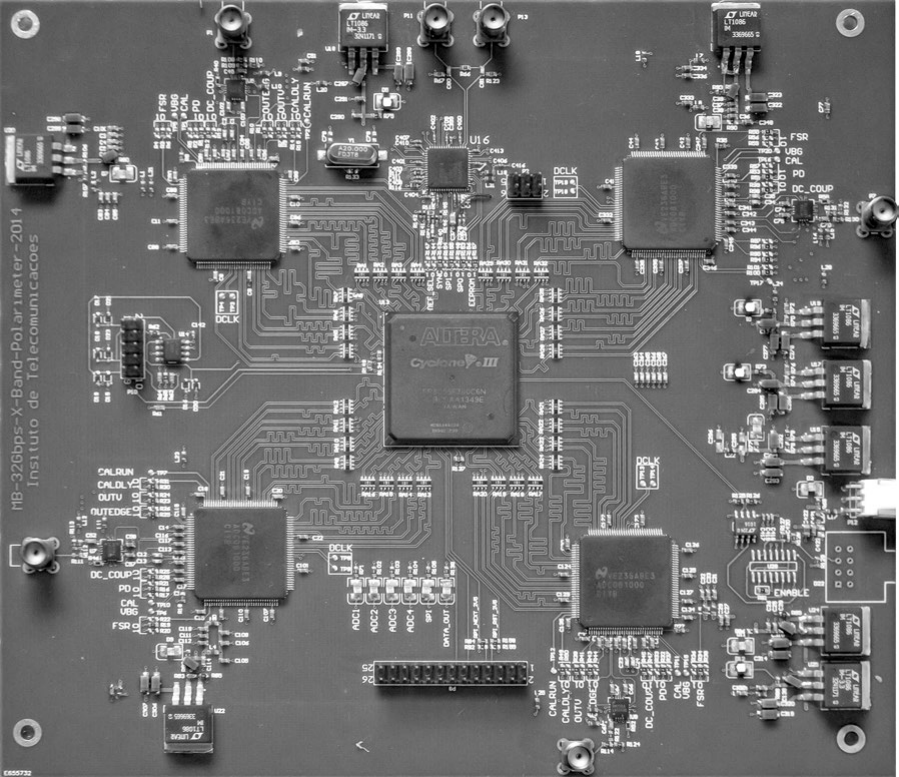
Correlator printed circuit board

The results presented below were obtained from the fabricated and assembled printed circuit board (PCB). The board with its assembled components is about 14 × 20 cm and is presented in Fig. 4.

The board has four layers in the following order (from top to bottom): Signal, Ground, Power and Signal. The main chip components observed in the highly symmetrical layout are the FPGA (Cyclone III – EP3C55F780C6), the ADCs (ADC081000) positioned at the FPGA corners, the differential ADC drivers (LMH6555) close to the ADC and the clock generator (AD9522-4). Between the two bottom ADCs is the general purpose input output (GPIO) bus connector. At the left side are the configuration devices of the FPGA (Active Serial) and one fiber link (ST connector near the border). At the rightmost side of the layout are the power supply voltage regulators.

The use of single ended FPGA pins requires their physical separation from the differential input pins. Detailed care was taken with the design of differential lines, separation, coupling, length equalization and termination, power supplies decoupling and signal integrity (Cao et al. 2011; Kim et al. 2010; Sharawi 2004).

As long as the connections between elements (I/O pin to register) remain short enough and with small length dispersion to stay within the clock sampling time window, it is possible to implement a standard synchronous pipeline architecture. The pins transmission directly to an input register helps to maximize the timing performance and allows faster setup times.

**Fig. 5:**
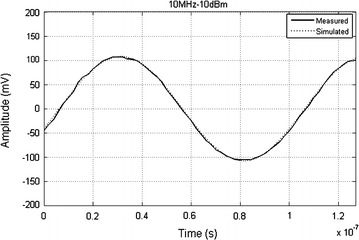
Acquisition of a sinusoid at 10 MHz: simulated versus acquired time series

The reconstruction of several input signal sinusoids with different amplitudes and frequencies were tested. However, we opted to only present in Fig. 5 the reconstruction of an acquired sinusoid with a power of −10 dBm and 10 MHz frequency.

The previous figure clearly demonstrates an excellent acquisition of the input sinusoids. The small variations of the acquired sinusoid points are due to an improper digitization of the first and/or second least significant bits due to inherent noise of the system.

The second test phase consisted on obtaining the Stokes parameters for the linear, circular and partial polarizations. The operation of the correlator is simulated at the laboratory by inserting several polarization scenarios at the correlator.

**Fig. 6:**
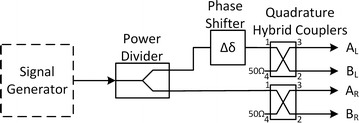
Polarimeter complete test set

**Fig. 7:**
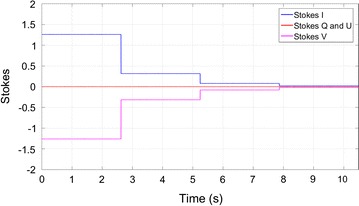
Linear left polarization Stokes parameters for four amplitude levels

The best approach here was to send the Stokes parameters time series, for later analysis, to a small computer using a GPIO interface. Three scenarios were considered: one for linear polarization, one for circular polarization and other for partial polarization. Two sinusoids with different amplitudes and frequencies for each polarization are created to mimic these polarizations Fig. 6. We opted to present the results of a circular polarization situation, that is, two sinusoids with the same amplitude and frequency and phase imbalance of π/2 radians. The power of the testing signal was manually switched during a few seconds through the levels of -12 dBm, -18 dBm, -24 dBm and -30 dBm. The test results are shown in Figs. 7 and in 8.

**Fig. 8:**
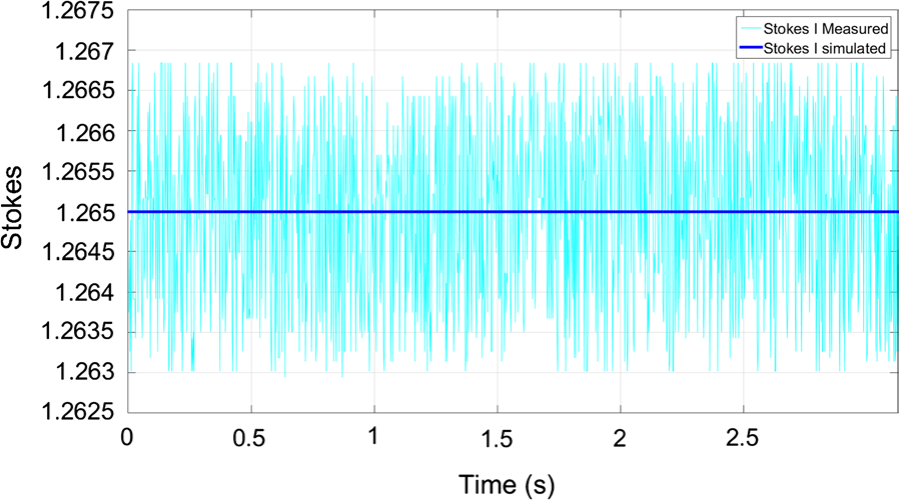
Linear left polarization Stokes I parameter: simulated versus measured

The time series shown corresponds to the Stokes parameter calculation considering the acquisition of two sinusoidal signals of 10 MHz described above at a rate of 1 GSps. The residual differences between the measured and simulated results are in line with the inaccuracy of the simulated amplitude sinusoid which corresponds to a standard deviation of 0.009, or a SNR of 34 dB.

The test to other polarizations (partial polarization) demonstrated a lower value of the standard deviation (to 0.01) proving that a correct operation of the cross correlation Stokes parameters (U and V). The tests consisted on receiving 5000 samples (for each Stokes parameter) from the correlator, each sample represents an average of the 65,536 accumulations performed in the correlator. Since each accumulation takes 2 s a sole test takes 10 s as presented in Fig. 7.

**Fig. 9:**
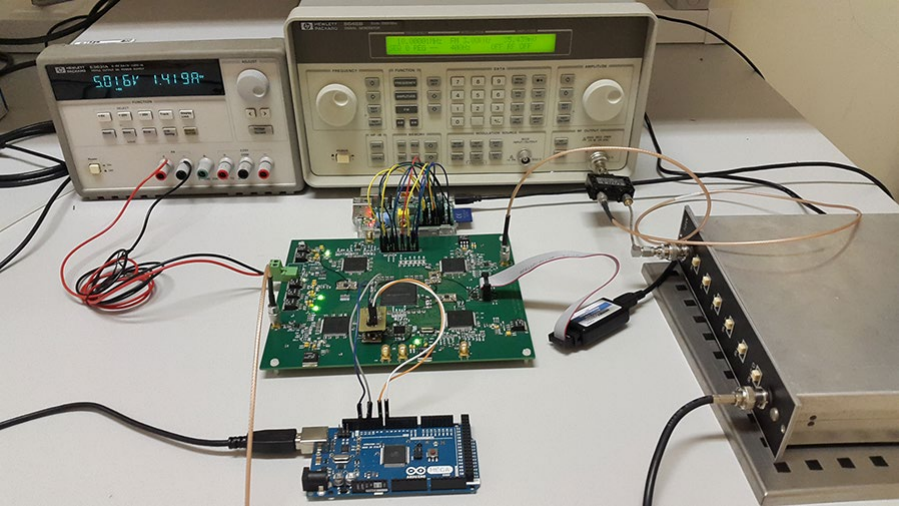
Layout of the polarimeter test bench

The layout of the test bench is presented in Fig. 9.

A summary of the used FPGA resources is presented in Table 2.

**Table 2:** Resources used by the FPGA

Device cyclone III FBGA 780 pins	EP3C55F780C6
Total logic elements	10,637/55,856 (19 %)
Total combinatory functions	6340/55,856 (11 %)
Dedicated logic registers	5273/55,856 (9 %)
Total registers	5337
Total memory bits	1032/239,6160 (<1 %)
Embedded multiplier 9-bit elements	256/312 (82 %)
Total PLLs	4 (100 %)

Finally, Table 3 summarizes the hardware price contributions for a prototype. This table confirms the possibility of a low to medium scale production for less than €1000.

**Table 3:** Estimated price for the correlator PCB

540 components in 4 layers	FR4 1.6 mm
PCB fabrication	315 €
PCB assembly	305 €
Components	755 €
Total price	1375 €

## Conclusions

The design of wide bandwidth digital receivers based on fast ADCs and FPGA data processing is important for RA applications requiring large integration time and where calibration procedures and receiver stability are mandatory. These digital designs require agile interference detection and cancellation, communication systems using spatial diversity and FPGAs smart coding.

The development of a cost effective 1 GHz bandwidth digital polarimeter required implementation of the VLSI Hardware Description Language (VHDL) code at the register transfer level with a high degree of parallelization. The device clock distribution is based on an external clock generator combined with FPGA PLL resources to drive all the system (FPGA and 4 ADCs) with adequate timing and high synchronization.

The achieved results prove the hypothesis of having a low range FPGA as a data processing unit operating at high frequency (500 MHz) without the use of dedicated high speed buffers [Multi Gigabit Transceiver (MGT), Rocket I/O] or external devices, such as serializers, that increase the price and complexity of the fabrication. The main goal is to operate the ADCs, FPGA and clock generator at its maximum limits. Indeed, this digital backend enables GEM to proceed towards acquisition of data over large sky areas in X-band with high sensitivity. As a corollary, projects with massive needs of digital processing subsystems like the SKA, may benefit from this approach since the main hardware characteristic of this high bandwidth design is the use of a conventional FPGA and off-the-shelf components, making the design affordable for projects requiring cost-effective digital processing.

## Abbreviations

GSM: global sky model
Gbps: giga bits per second
GSps: giga sample per second
ADC: analogic to digital converter
FPGA: field programmable gate array
GEM: galactic emission mapping
VHDL: VLSI hardware description language
RA: radio astronomy
SKA: square kilometer array
LOFAR: low-frequency array
MWA: murchison widefield array
MeerKAT: Karoo Array Telescope
ASKAP: Australian square kilometer array pathfinder
RF: radio frequency
IF: intermediate frequency
RFI: radio frequency interference
CASPER: collaboration for astronomy signal processing and electronic research
CMB: cosmic microwave background
CBASS: C-band all sky survey
HPBW: half-power beam width
OMT: orthomode transducer
LHCP: left hand circular polarizations
RHCP: right hand circular polarizations
LNA: low noise amplifier
pHEMT: pseudomorphic high electron mobility transistor
mHEMT: metamorphic high electron mobility transistor
LPF: low pass filter
PLL: phase lock loop
MSps: mega samples per second
Kbps: kilo bytes per second
ACC: accumulator
MUX: multiplexer
FIFO: first in first out
LVDS: low voltage differential signaling
BGA: ball grid array
I/O: inputs and outputs
PCB: printed circuit board
GPIO: general purpose input/output
MGT: multi gigabit transceiver
